# The O_2_-sensitive brain stem, hyperoxic hyperventilation, and CNS oxygen toxicity

**DOI:** 10.3389/fphys.2022.921470

**Published:** 2022-07-26

**Authors:** Jay B. Dean, Nicole M. Stavitzski

**Affiliations:** Department of Molecular Pharmacology and Physiology, University of South Florida, Tampa, FL, United States

**Keywords:** hyperoxia, O2-sensing, CO2-chemosensitive, cardiorespiration, seizure, hyperbaric oxygen therapy, undersea medicine

## Abstract

Central nervous system oxygen toxicity (CNS-OT) is a complex disorder that presents, initially, as a sequence of cardio-respiratory abnormalities and nonconvulsive signs and symptoms (S/Sx) of brain stem origin that culminate in generalized seizures, loss of consciousness, and postictal cardiogenic pulmonary edema. The risk of CNS-OT and its antecedent “early toxic indications” are what limits the use of hyperbaric oxygen (HBO_2_) in hyperbaric and undersea medicine. The purpose of this review is to illustrate, based on animal research, how the temporal pattern of abnormal brain stem responses that precedes an “oxtox hit” provides researchers a window into the early neurological events underlying seizure genesis. Specifically, we focus on the phenomenon of hyperoxic hyperventilation, and the medullary neurons presumed to contribute in large part to this paradoxical respiratory response; neurons in the caudal Solitary complex (cSC) of the dorsomedial medulla, including putative CO_2_ chemoreceptor neurons. The electrophysiological and redox properties of O_2_-/CO_2_-sensitive cSC neurons identified in rat brain slice experiments are summarized. Additionally, evidence is summarized that supports the working hypothesis that seizure genesis originates in subcortical areas and involves cardio-respiratory centers and cranial nerve nuclei in the hind brain (brainstem and cerebellum) based on, respectively, the complex temporal pattern of abnormal cardio-respiratory responses and various nonconvulsive S/Sx that precede seizures during exposure to HBO_2_.

“*. . .oxygen pressure in the mammalian CNS is maintained at a level which is sufficiently high to ensure undisturbed function of brain cells and sufficiently low to minimize generation of free radicals*” (Erecińska and Silver 2001).

“*Interest in studies of oxygen toxicity is not limited to those who contemplate the exotic environments of sea and space. Such studies may lead to clarification of oxygen effects in normal man at sea level and in patients with cardio-respiratory and other diseases*” (Editor 1966)

## Hyperoxia, an unnatural but commonly encountered condition

The brain stem is acknowledged as the primary site for integrated, coordinated control of mammalian cardio-respiration; for example, respiratory rhythmogenesis and establishing breathing pattern (Ramirez and Baertsch 2018); regulation of blood pressure *via* regulation of heart rate, stroke volume, and total peripheral resistance (Benarroch 2018); central CO_2_-chemoreception (Guyenet et al., 2010; Dean 2011; Dean and Putnam 2010) and central integration of peripheral chemoreceptor (O_2_, CO_2_) and baroreceptor afferent inputs (Accorsi-Mendonca and Machado 2013). Typically, physiologists discuss cardio-respiratory control in terms of brain stem chemo-reflexes activated during perturbations in arterial blood gases and brain cerebrospinal fluid and extra-/intracellular fluids that cause hypoxemia (Powell et al., 1998), chronic intermittent hypoxia and reoxygenation (Prabhakar et al., 2001), and systemic acute and chronic hypercapnic (respiratory) acidosis (Putnam et al., 2005; Nattie and Li 2009; Nattie and Li 2010; Dean 2011).

But what about exposure to hyperoxia? How does exposure to hyperoxia and activation of redox signaling and possibly oxidative stress affect brain stem neurons and respiration? The strictest definition of hyperoxic ventilation is inspiring a gas mixture having a partial pressure of oxygen (P_I_O_2_) greater than 159.6 mm Hg or Torr (Dean et al., 2003); that is, greater than the product of barometric pressure at sea level (P_B_ = 760 mm Hg or Torr) and the fractional concentration of inspired oxygen in air ( $F_{I}O_{2}=0.21$ ), $P_{I}O_{2}\text{>}\left(760\;\mathrm{Torr}\text{×0}\text{.}21\;{\text{O}}_{2}\right)\text{>}159\text{.}6\;\mathrm{Torr}\;O_{2}$ .

If one is working in pressure units of atmospheres absolute (ATA), then physiologists define a hyperoxic atmosphere as, $P_{I}O_{2}\text{>}\left(1\;\mathrm{ATA}\text{×0}{\text{.}21\text{O}}_{2}\right)\text{>0}\text{.}21\;\mathrm{ATA}\;O_{2}$ .^1^

Hyperoxic ventilation occurs in pulmonary medicine (Fleetham et al., 1982; Branson 2018), hyperbaric oxygen therapy (HBOT) (Neuman and Thom 2008), and in occupations conducted in extreme environments that are medically supported by the hyperbaric, undersea, and aerospace communities (Davis 1995; Clarke 2008; Moon 2014; Manning 2016). The physiologically relevant continuum of hyperoxia experienced by humans begins at >0.2 up to 1 ATA O_2_ (normobaric hyperoxia, NBO_2_) and continues up to 3.0 ATA O_2_ (hyperbaric oxygen, HBO_2_), where HBO_2_ is defined as any P_I_O_2_ > 1 ATA O_2_. The use of HBO_2_ is limited by the risk of central nervous system oxygen toxicity (CNS-OT); however, if the dose of oxygen is large enough (P_I_O_2_ × minutes) then the risk of early symptoms of pulmonary oxygen toxicity become a concern (Jackson 1985). The hallmark sign of CNS-OT is generalized seizures followed by cardiogenic pulmonary edema (Demchenko et al., 2007; Demchenko et al., 2011).

Currently, there are fourteen approved indications for HBOT in the United States that include, for example, carbon monoxide poisoning, aeroembolism, and problematic wound healing. Examples of wound healing that respond to HBOT include crush injury, compartment syndrome and other acute traumatic ischemias (UHMS 2018). A single session of HBOT typically uses from 1.4 to 3.0 ATA O_2_ over multiple 20–30-min exposures that are interspersed with 5–10-min air breaks to interrupt the cumulative effects of hyperoxia and reduce the risk for CNS-OT (UHMS 2018). In undersea medicine, the primary use of HBO_2_ is the closed-circuit oxygen rebreather, a specialized underwater breathing apparatus whereby the diver breathes 100% oxygen, and all gases are contained and recirculated within the rebreathing apparatus. Using pure oxygen averts the buildup of nitrogen in the diver that occurs when breathing air and allows all the gas carried by the diver to be used for their metabolic needs. And, importantly for warfare operations, the closed-circuit O_2_ rebreather adds a stealth aspect in that no bubbles are produced during diver exhalation. All the diver’s exhaled gas is recirculated, and exhaled CO_2_ is chemically removed while O_2_ is replenished from a tank carried by the diver. In addition, the closed-circuit O_2_ rebreather, compared to the open-circuit, self-contained underwater breathing apparatus, extends diver operation time and is smaller and weighs less; however, it introduces the added risk of CNS-OT the deeper one descends (USN 2016). When diving with a closed-circuit O_2_ rebreather, the U. S. Navy guidelines limit O_2_ exposure at 50 fsw (2.5 ATA) to 10 min to reduce the risk of developing CNS-OT (USN 2016). The risk of CNS-OT is greater while submerged in water than during HBOT in a dry chamber due to reflexive changes in blood flow activated by immersion that increase cerebral blood flow (Pendergast et al., 2015) and thus brain PO_2_ relative in P_I_O_2_ (Demchenko et al., 2005).

Animal models used to study mechanisms and mitigation of CNS-OT, however, use higher levels of HBO_2_ ranging from 4 to 5 ATA (unanesthetized animals) (Pilla et al., 2013; Held et al., 2014; Posada-Quintero et al., 2022) and 6 ATA O_2_ (anesthetized animals) (Demchenko et al., 2012). The rationale for using a higher level of HBO_2_ in the rodent model is to accelerate onset of neurological Sz while avoiding the confounding effects of pulmonary oxygen toxicity, which take longer to occur (Jackson 1985; Ciarlone et al., 2019). Recently, in a preliminary study, we determined that adding 1.75–2.5% CO_2_ to O_2_ and compressing male Sprague-Dawley rats to only P_B_ = 3 ATA (∼2.9 ATA O_2_, balance CO_2_) induces seizures with a latency similar to that of 100% O_2_ breathed at 5 ATA (Dean et al., 2022). In contrast, others have reported that exposure to 3 ATA 100% O_2_ takes hours to produce Sz in rodents, if at all, and protracted exposures to this level of HBO_2_ induce early symptoms of pulmonary oxygen toxicity (pulmonary congestion, irritation, and atelectasis). For example, mice exposed to 3 ATA O_2_ for 6 h did not develop CNS-OT but did develop tachypnea (Demchenko et al., 2008). The reasons that CO_2_-retention accelerates CNS-OT is it causes cerebral vasodilation and increases brain tissue PO_2_ relative to P_I_O_2_ (Jamieson and Van den Brenk 1963), and enhances production of reactive oxygen species (ROS) and reactive nitrogen species (RNS) relative to normocapnic hyperoxia (Dean 2010). We’ll refer to ROS and RNS together as RONS for the remainder of this article. Thus, it appears that lower levels of HBO_2_ can be used in conjunction with modest levels of CO_2_ retention in animal models to study mechanisms and mitigation of CNS-OT. Including CO_2_-retention with HBO_2_ is an acceptable protocol given that CO_2_ retention is a serious problem in undersea medicine that exacerbates the risk for CNS-OT (Ciarlone et al., 2019).

Hyperoxia, additionally, is used by neuroscientists that employ reduced rodent brain tissue preparations to study, among other things, the neural control of respiration (Okada et al., 1993; Paton 1996; Wilson et al., 2001). With very few exceptions (Dean et al., 2003; Ciarlone et al., 2019), however, the effects of hours-long exposure to hyperoxia and uninterrupted activation of redox and nitrosative signaling mechanisms in reduced mCNS preparations are never considered as a confounding factor when interpreting neurophysiological data. This is potentially problematic given the ubiquitous role of redox signaling in the mCNS (Dröge 2002) and reports that as few as one to three bouts of increased oxygen delivered from a level of hypoxia (reoxygenation) and control O_2_ induces long lasting changes in neural excitability (Garcia III et al., 2010) and respiratory drive (Fuller et al., 2000). As discussed elsewhere (Dean et al., 2003; Ciarlone et al., 2019), this raises the following question—what do hours of exposure to hyperoxia do to neuronal function and what are the underlying mechanisms activated during protracted exposure to an O_2_-enriched “control” environment? In their paper discussing the preparative methods for brain slices, Aitken and colleagues were ambivalent, nay lackadaisical, on the issue of using 95% O_2_ to aerate brain slice nutrient media as the gold-standard control condition,

“. . . it is a standard Krebs bicarbonate buffer, pregassed with 5% CO_2_ and 95% O_2_. . . maybe we use too much O_2_. I don’t know” (Aitken et al., 1995).

We would propose that neurophysiologists should want to know the answer to this question. By chasing the answer down the research community will gain new insights on O_2_ and redox signaling in the mCNS in the context of health and disease and refine the baseline conditions used in popular reduced mCNS preparations. Hopefully, one of the outcomes of reading this article are new ideas for research on the use of oxygen in neurophysiology that “…may lead to clarification of oxygen effects in normal man at sea level and in patients with cardio-respiratory and other diseases” (Editor 1966).

Given the prevalent occurrence of hyperoxic ventilation (*in vivo*) and hyperoxic conditions (*in vitro*) as described above, it is worthwhile examining what hyperoxia does to brain stem neurons and the systems they control, including respiration. This is an interesting issue because hyperoxia, unlike exposure to hypoxia, is an *unnatural condition* that rarely occurs naturally in life. The only means by which to make oneself hyperoxemic effortlessly is to visit the Dead Sea and experience “natural oxygen enrichment” (Kramer and Godfrey 1996) at the local elevation of ∼1,400 feet *below* sea level where barometric pressure (P_B_) > 1 ATA and P_I_O_2_ is > 160 Torr. Patients suffering COPD that normally reside at moderate or high altitude (Jerusalem) show marked cardio-pulmonary improvement in arterial oxygenation and exercise capacity during a 3 weeks visit to the Dead Sea (Kramer et al., 1998).

For those of us who don’t visit the Dead Sea frequently, the only other way to become hyperoxic is through some combination of the following human-made interventions: 1) increase the F_I_O_2_ in the breathing gas mixture (i.e., O_2_-enriched atmosphere); 2) increase the P_B_ of the air or O_2_-enriched gas mixture through compression; or 3) both. Of course, the logistics of breathing an O_2_-enriched artificial atmosphere require pumps to pressurize the gas mixture, storage of the compressed O_2_-enriched gas in steel cylinders that are then vented by controlled means through high- and low-pressure gas flow regulators to the patient or subject wearing a nasal cannula or oronasal mask (Sheffield et al., 1977; Nolan et al., 1993). Alternatively, hyperoxic ventilation takes place using hardware of even greater complexity; for example, breathing air or O_2_-enriched gas mixture directly from an enclosed, contained atmosphere while hermetically sealed in one of the following vehicles or suits: hyperbaric chamber (UHMS 2018), pressurized work caisson (Kindwall 1993; Kindwall 1997), underwater habitat (Strewea et al., 2015; Koutnik et al., 2021), submarine (Mahon et al., 2009; Schoenwiesner et al., 2020), space vehicle (Webb and Pilmanis 1998), or pressurized flight suit (Gradwell 2006).

Our point is this. The mCNS did not evolve to function normally under hyperoxic conditions. Case in point; the brain exhibits cardio-respiratory chemoreflexes that restore systemic oxygenation from hypoxemia caused by alveolar hypoventilation, pulmonary disease, and ascent to higher elevations. By contrast, the respiratory response of the mCNS to hyperoxia is paradoxical; that is, hyperoxic hyperventilation (Dean et al., 2004). Increasing alveolar ventilation, if anything, will only further increase brain PO_2_. The acronym, HO_2_VR (hyperoxic ventilatory response), is introduced here to describe this less recognized form of alveolar hyperventilation from the other two types of alveolar hyperventilatory responses, popularly referred to as HVR (hypoxic ventilatory response) and HCVR (hypercapnic ventilatory response).

The purpose of the remainder of this review is to focus on the phenomenon of hyperoxic hyperventilation, and the medullary neurons presumed to contribute in large part to this paradoxical respiratory response. Hyperoxic hyperventilation and other early toxic indications of brain stem origin that precede CNS-OT (*Integrated cardio-respiratory responses to hyperoxia*) provide us a window into the early neurological events underlying seizure genesis when breathing HBO_2_.

## Integrated cardio-respiratory responses to hyperoxia

The respiratory gases, O_2_ and CO_2_, are powerful stimulants of respiration in mammals; exposure to hypoxia and/or hypercapnia stimulate breathing to restore, respectively, systemic oxygenation and PCO_2_/pH homeostasis (West 2012). Hyperoxia, by contrast, activates a compound three-phase respiratory response that is influenced by the dose of oxygen (i.e., the oxygen concentration product, P_I_O_2_ × duration of hyperoxia) and the ensuing end-tidal CO_2_ (Dean et al., 2004). Exposure to NBO_2_ (100% O_2_ at P_B_ = 1 ATA) usually inhibits respiration through inhibition of the carotid body chemoreceptors. Known as “chemical denervation” or “physiological chemodenervation” of the peripheral chemoreceptors, this technique was introduced in 1947 (Dripps and Comroe 1947) and has been widely used ever since in cardio-respiratory control research to effectively silence afferent input from the peripheral hypoxia/hypercapnia chemoreceptors during experimental manipulations; for example (Becker et al., 1996; Wilkinson et al., 1997; Gozal 1998). The popular interpretation when using hyperoxic hypercapnia under *in vivo* conditions is that any change in breathing is related to “chemical denervation” of the peripheral chemoreceptors and activation of the central chemoreceptors. What is often unrecognized, however, is the important caveat stated in 1947 by Dripps and Comroe,

“It must be remembered that a stimulant effect of oxygen which tends to increase respiration may be acting simultaneously to limit the extent of this immediate depression of minute ventilation.”^2^

**FIGURE 1:**
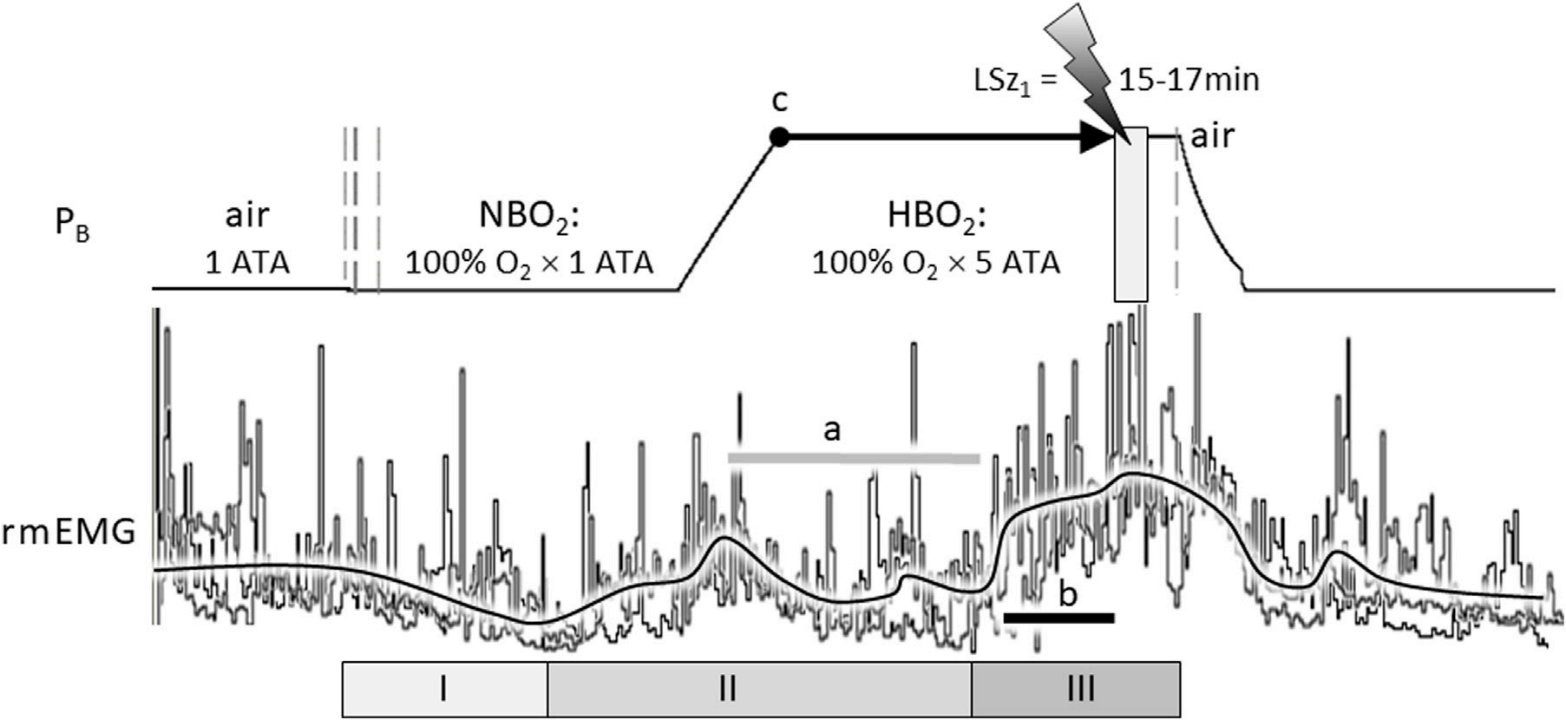
The compound hyperoxic ventilatory response (HO_2_VR) has three distinct phases (I-III). These experiments were done in a male Sprague-Dawley rat that was exposed to hyperbaric oxygen (HBO_2_: 5 ATA) three times, once every 7 days, until onset of seizure (lightning bolt). The latency time to first seizure (LSz_1_) was measured from time reaching maximum depth (5 ATA, time = 0, indicated by “c”) until onset of increased cortical electroencephalogram activity and visible seizure (not shown). In this animal, LSz_1_ ranged from 15 to 17min over 3 “dives”; the grey box at the lightning bolt defines the range of LSz_1_ values for 3 dives. Phase I HO_2_VR—breathing decreases and is attributed to inhibition of peripheral low O_2_ chemoreceptions (i.e., peripheral chemoreceptor physiological denervation). Phase I, if it occurs, is transient and lasts a few minutes. Phase II HO_2_VR—continued breathing of NBO_2_ followed by HBO_2_ stimulates respiration; however, alveolar hyperventilation blows off CO_2_ and blunts or attenuates breathing in the second half of phase II (horizontal grey line “a”). Phase III HO_2_VR—continued breathing of HBO_2_ builds up oxidative stress centrally and stimulates neurons, presumably O_2_-/RONS-/CO_2_-sensitive neurons in the cSC and other regions. In this example, phase III began from ∼5 to 7 min prior to Sz (length of black line “b” in phase III is equal to 5 min). The vertical dashed lines on the barometric pressure (P_B_) trace (left) indicate when the rat’s breathing atmosphere was changed from 1 ATA air to 100% O_2_ at room pressure (NBO_2_) or, after seizure, from 100% O_2_ at 5 ATA (HBO_2_) to air (right) followed by decompression. The three sets of respiratory traces (rmEMG, respiratory muscle electromyogram) were superimposed and aligned on the y-axis to emphasize the consistent temporal pattern of the HO_2_VR over all 3 dives. Likewise, the black line averaging the breathing responses was drawn manually by eye to emphasize the three phases of the HO_2_VR. Refer to the text for further details on the HO_2_VR. Figure 1 was adapted from (Pilla et al., 2013).

Indeed, the stimulating effect of excess oxygen on respiration has been reported in humans and animals, including human infants (Davi et al., 1980; Rigatto et al., 1982) and adults (Dautrebande and Haldane 1921; Dripps and Comroe 1947; Lambertsen et al., 1953; Georgopoulos et al., 1989; Becker et al., 1996; Ren et al., 2000) and animal models, including rat (Pilla et al., 2013; Stavitzski et al., 2022), cat (Gautier et al., 1986; Mokashi and Lahiri 1991), and dog (Watt et al., 1943). For example, Figure 1 illustrates the stimulatory effect of breathing hyperoxia on the ventilatory response of an unanesthetized, freely behaving adult male Sprague Dawley rat during exposure to 1 ATA air, 1 ATA O_2_ (NBO_2_: 100% O_2_ at room pressure) and 5 ATA O_2_ (HBO_2_: 100% O_2_ at 132 feet of sea water, fsw). Respiration was measured using a pair of radio telemetric electrodes embedded in the chest wall at the insertion of the diaphragm (rmEMG, respiratory muscle electromyogram). The experiment was repeated in the same animal three times, once every 7 days over 2 weeks, until onset of generalized seizures (Sz); i.e., CNS-OT. Three phases of the HO_2_VR are clear. In phase I, minute ventilation is either unchanged (Miller and Tenney 1975; Gautier et al., 1986; Becker et al., 1996; Ren et al., 2000) or decreases (Watt et al., 1943; Dripps and Comroe 1947), as in this example, and is interpreted as the period of carotid body physiological denervation. Inhibition of the peripheral chemoreceptors, however, is transient and continued breathing of NBO_2_ and/or HBO_2_ induces hyperventilation, phase II, during which arterial and end-tidal PCO_2_ decrease (Becker et al., 1996; Ren et al., 2000). Thus, central hyperoxia initially stimulates breathing, which lowers systemic CO_2_ and inhibits central CO_2_-chemoreceptors (a). Not surprisingly, the magnitude of the initial hyperventilation is dependent on the level of end-tidal CO_2_, being significantly larger during exposure to isocapnic hyperoxia but blunted and potentially masked during exposure to poikilocapnic hyperoxia (Georgopoulos et al., 1989; Becker et al., 1996; Ren et al., 2000). This may explain why phase II of the HO_2_VR response was not always reported by investigators using poikilocapnic hyperoxia under normobaric conditions.

Continued breathing of HBO_2_ further increases levels of oxidative stimuli in the brain (Thom 2009) that, we propose, eventually activates phase III, which begins ≤8 min (Pilla et al., 2013) to ≤15 min (Stavitzski et al., 2022) prior to Sz. Accumulating evidence supports the use of phase III HO_2_VR as an early warning “physio-marker” of an impending Sz in CNS-OT alone (Pilla et al., 2013) and in conjunction with acute exogenous ketone therapy which delays Sz genesis by over 300% (Stavitzski et al., 2021).

Neural control of ventilation, however, is integrated and coordinated at the level of the brain stem with cardiovascular control of heart rate, stroke volume, and total peripheral resistance, the product of which determines arterial blood pressure (Demchenko et al., 2011; Benarroch 2018). Initially, during the safe latent period of breathing HBO_2_, there is further evidence that autonomic and brain stem control centers are stimulated by hyperoxia. In anesthetized animals, exposure to HBO_2_ results in a parasympathetic response comprised of decreased heart rate, cardiac output, blood pressure, and sympathetic tone (Demchenko et al., 2012; Gasier et al., 2018). Cerebral vasoconstriction initially attenuates the rise in brain tissue PO_2_ and is fundamentally important for determining the length of the safe latent period of HBO_2_ ventilation (Demchenko et al., 2000a; Demchenko et al., 2000b; Demchenko et al., 2001; Atochin, Demchenko et al., 2003; Demchenko et al., 2005; Demchenko et al., 2010). In unanesthetized animals, the bradycardia is ongoing throughout exposure to HBO_2_ (Pilla et al., 2013; Stavitzski et al., 2022).

Continued breathing of HBO_2_ activates increased sympathetic outflow leading to hyperoxic hyperventilation (phase II) and hypertension in anesthetized animals (Pilla et al., 2013; Gasier et al., 2015; Gasier et al., 2018). The shift in autonomic output is due in part to reflexive compensation for pressure-induced activation of the arterial baroreceptor response (Demchenko et al., 2014). Additional evidence of generalized autonomic activation include cooling of deep body temperature (Puglia et al., 1974; Butler and Thalmann 1986; Fenton et al., 1993; Shmalberg et al., 2015) and increased electrodermal resistance (Posada-Quintero et al., 2022). As sympathetic neural drive increases there is a central buildup in nitric oxide (⋅NO) in the mCNS that induces cerebral vasodilation causing a rapid surge in regional cerebral blood flow (rCBF) and thus brain tissue PO_2_ and RONS production and, ultimately, seizures (Demchenko et al., 2001; Demchenko et al., 2003; Demchenko et al., 2007). Once sustaining a “CNS oxtox hit” or Sz, the upwelling in autonomic sympathetic activity and catecholamine release continues undiminished and depresses left ventricular function and increases arterial and pulmonary vascular pressure resulting in cardiogenic pulmonary edema (Demchenko et al., 2007; Demchenko et al., 2011).

The temporal sequence of abnormal respiratory responses explains prior accounts of abnormal breathing patterns that precede loss of consciousness when sustaining a “CNS oxtox hit” while diving. During World War II, Dr. Christian (Chris) J. Lambertsen^3^ (1917–2011), who is acknowledged by the U. S. Navy as the “father of frogmen”, developed the U. S. military’s first generation closed-circuit O_2_ rebreather for use in clandestine diving military operations. He called it the “rebreathing apparatus” and later rechristened it the LARU for Lambertsen Amphibious Rebreather Unit (Vann 2004). The story goes that while training military diving instructors how to use his O_2_ rebreathing apparatus for rescue and recovery operations at sea, Chris, distracted, floated deeper than he should have, suspended in 100 fsw breathing 100% O_2_ (P_I_O_2_ = 4 ATA O_2_). As mentioned above, immersion in water redistributes the body’s circulation, including increased rCBF and thus increased brain oxygenation and RONS production that cause seizures to occur faster (Pendergast et al., 2015; Ciarlone et al., 2019). For example, at 90 fsw the latency time to first seizure (LSz_1_) is ∼128 min in a dry chamber and only ∼41 min in a wet chamber (Donald 1947a; Donald 1947b; Donald 1992). In Chris Lambertsen’s case,

“…after drifting deeper than intended, he found himself at 100 feet (30 m) with a fluttering diaphragm. His last action before an O_2_ seizure was to inflate his breathing bags in order to become positively buoyant. He awoke with a buzzing in his head, staring into the eyes of a Navy chaplain” (Vann 2004).

Not all divers who experience CNS-OT underwater are as fortunate as Chris Lambertsen was that day (Lawrence 1996), which is why the U. S. Navy places strict limits on HBO_2_ exposure (USN 2016; Ciarlone et al., 2019). The risk of suffering CNS-OT while submerged is also why the diving community continues pursuing identification of early physio-markers and therapeutic interventions to reduce the threat of CNS-OT; reviewed in (Ciarlone et al., 2019).

## Hyperoxia and the brain stem

### Does the brain stem function as a site of Sz genesis for CNS-OT?

**FIGURE 2:**
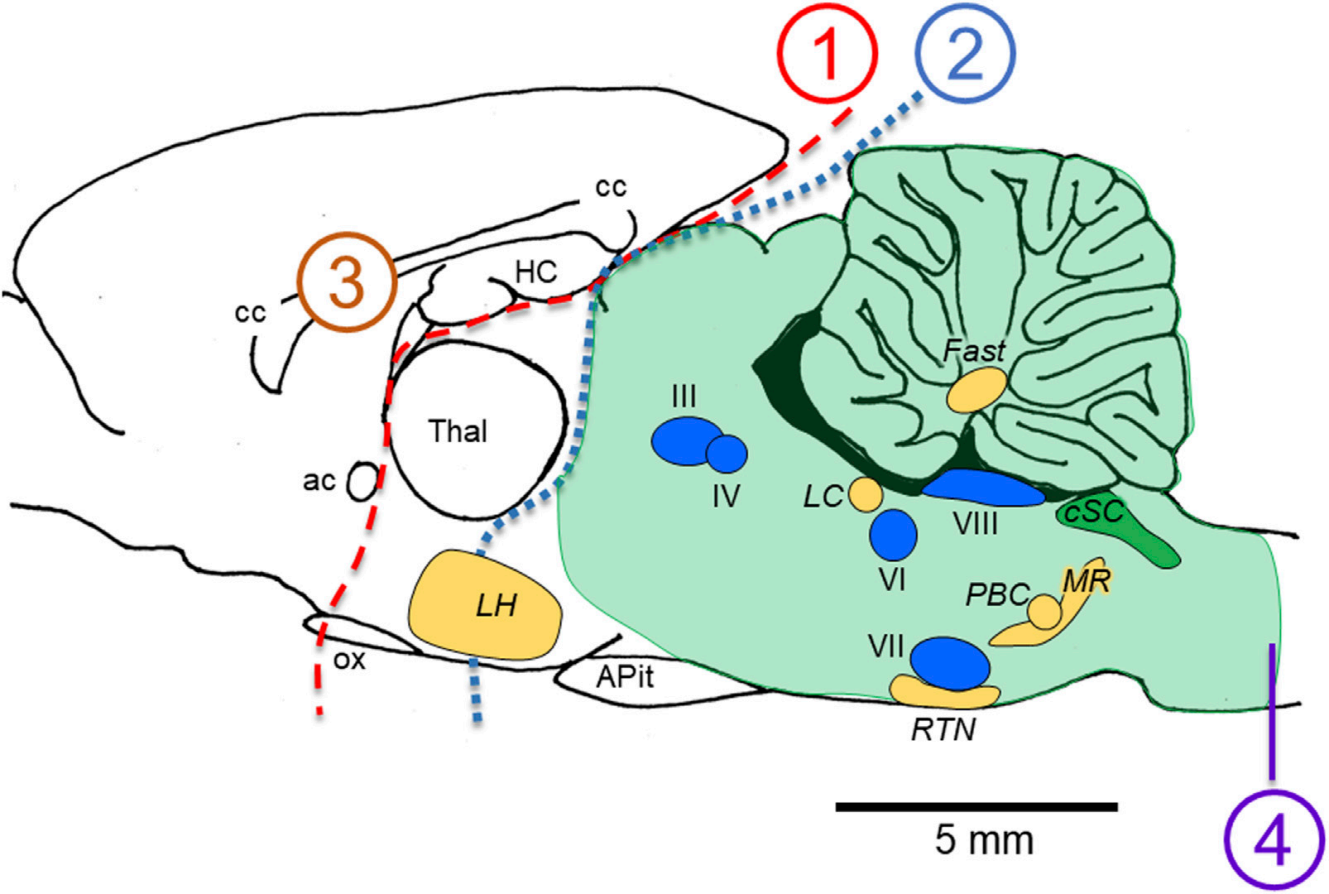
Locations of lesions in animal models that do not (1–3) versus do (4) abolish the seizures of CNS-OT. Removing the forebrain (lesions 1 and 2) and bisecting the corpus callosum (lesion 3) do not abolish seizure genesis when breathing HBO_2_. A medullary pyramidal tractotomy does abolish seizure, however (lesion 4). Thus, the critical areas involved in seizure genesis must reside between lesions 2 and 4; that is, in the hind brain; i.e., brain stem plus cerebellum (light green shaded region). Nuclei postulated to function as “oxtox trigger nuclei” based on the early abnormal cardio-respiratory responses and nonconvulsive S/Sx that precede HBO_2_-seizures are indicated, including CO_2_-chemosensitive areas (golden yellow nuclei) and cranial nerve nuclei (blue nuclei). While neurons in CO_2_-chemosenitive areas are reported to be stimulated during exposure to hypercapnic acidosis, to date, only CO_2_-sensitive neurons in the cSC (green nuclei) are reported to be stimulated by exposure to hyperoxia and chemical oxidants in control O_2_. CO_2_-excited neurons in the other chemosensitive areas of the mCNS have not been studied yet to determine their sensitivity to increased oxygenation and cellular oxidation. The sagittal view presented is 0.40 mm lateral to midline in the rat brain, which was adapted from Figure 164 in the brain atlas of Paxinos and Watson (Paxinos and Watson 2007). The relative locations of cranial nerve nuclei III, IV, VI, VII, VIII and X and chemosensitive nuclei indicated come from sagittal views passing 0.18 through 2.4 mm lateral to midline (Figures 163–171 in the rat brain atlas). Recall that CN X is the dorsal motor nucleus of vagus, which with the nucleus tractus solitarius comprises the cSC. The purpose of the Figure 2 is to emphasize, based on lesion studies, the region of the mCNS that likely contains neurons involved in seizure genesis in CNS-OT; that is, the rostro-caudal distribution of nuclei postulated to function as “oxtox trigger nuclei”. Their locations presented here do not to accurately convey their true medial-to-lateral distributions. Abbreviations used: III, oculomotor nucleus; IV, trochlear nucleus; VI, abducens nucleus; VII, facial nucleus; VIII, vestibular-cochlear nuclei; ac, anterior commissure; APit, anterior pituitary; cc, corpus callosum; cSC, caudal Solitary Complex (= NTS and DMNV/CN X); Fast, fastigial nucleus of the cerebellum; HC, hippocampus; LC, locus coeruleus; LH, lateral hypothalamus; MR, medullary raphe; ox, optic chiasm; PBC, pre-Bötzinger Complex; RTN, retrotrapezoid nucleus; Thal, thalamus.

The temporal complexity of the abnormal integrated cardio-respiratory responses observed during exposure to HBO_2_ is one of the main observations contributing to our working hypothesis that brain stem neurons are stimulated throughout most of the “O_2_-dive”, culminating in the neurological explosions that are manifested as generalized Sz. We (Ciarlone et al., 2019) and others (Bitterman 2004) have noted that the generalized seizures of CNS-OT likely do not originate in the cerebral cortex. Possible reasons for this are as follows. First, as illustrated in Figure 2, removal of the forebrain (transections 1 or 2) (Bean and Rottschafer 1938; Rucci et al., 1966) or bisecting the corpus callosum (transection 3) (Gowdey et al., 1965) does not block Sz genesis during exposure to HBO_2_; however, bisecting the medullary pyramids does abolish HBO_2_-induced Sz (transection 4) (Batini et al., 1954; Harel et al., 1969). Hence, the critical region for Sz genesis in CNS-OT must lie below the cerebral cortex (lesion 2), rostral to the medullary pyramids (lesion 4), and include the hind brain (i.e., brain stem and cerebellum, which are delineated by the shaded green area). This idea is further supported by observations that not all animals implanted with cortical electroencephalogram leads reveal increased neural activity prior to or during the behavioral Sz (Bitterman 2004; Hinojo et al., 2017; Ciarlone et al., 2019). The few attempts to measure neural activity concurrently in multiple sites of the mCNS in anesthetized animals has, to date, revealed no consistent temporal pattern of neural activation (Cohn and Gersh 1945; Bertharion and Barthelemy 1964; Harel et al., 1969).

Second, Gasier et al. (Gasier et al., 2015) propose that regions of the mCNS where the earliest and largest increases in rCBF occur are important in Sz genesis. They found that rCBF and thus neural tissue PO_2_ and RONS production, increased in subcortical areas, followed by increased blood flow in the cerebral cortex and cerebellum. Subcortical regions that had the highest regional blood flow prior to Sz included the striatum (basal ganglia), hippocampal cortex (limbic system), hypothalamus (diencephalon), and nucleus tractus solitarius (NTS; medulla oblongata). Recall that the NTS is the dorsal half of the cSC, the O_2_-sensitivity which is discussed in *The O_2_-CO_2_ “gasophilic” cSC*).

Third, in addition to the abnormal cardio-respiratory responses outlined above, a variety of nonconvulsive S/Sx precede Sz in humans that are associated with brain stem nuclei; specifically cranial nerve (CN) nuclei III, IV, VI, VII and VIII. Nonconvulsive S/Sx are accepted by the hyperbaric and undersea medical communities as early toxic indications of CNS-OT (Donald 1947a; Donald 1947b; Yarbrough et al., 1947; Vann 1988; Donald 1992). To easily recall these early toxic indications, the U. S. Navy uses the mnemonic VENTID as follows (USN 2016): “V” is for blurred and tunnel Vision; “E” is for Ears and tinnitus; “N” is for Nausea and vomiting; “T” is for Twitching or Tingling of peripheral and facial muscles; “I” is for Irritability or changes in mood; and “D” is for Dizziness. All VENTID indications do not occur concurrently and in some cases seizures in HBO_2_ occur without any early nonconvulsive S/Sx warning (Yarbrough et al., 1947; Balentine 1982; Donald 1992). The fact that these specific S/Sz occur at all, however, provides insight as to what parts of the mCNS are likely activated prior to expression of a motor Sz. Five of the six early toxic indications are likely associated with increased electrical signaling by brain stem CN nuclei (Ciarlone et al., 2019). For example, visual auras, hallucinations, and visual disturbances (“V” in VENTID) imply involvement of the CNs controlling vision and eye movement (CN III, IV, VI: Oculomotor, Trochlear, Abducens nerves, and associated brain stem and midbrain nuclei). Auditory auras intimate involvement of the auditory receptors (“E” for ears) and activation of CN VIII (Vestibulocochlear nerve), whereas nausea (“N”) and vomiting also indicate irritation of CN VIII and stimulation of the vomit center in the brain stem (Carpenter and Sutin 1983; Borison et al., 1984). Twitching of facial muscles (“T”), including the lips, implies stimulation of CN VII (Facial nerve). Dizziness (“D”) or vertigo also indicates involvement of CN VIII (Vestibulocochlear nerve) and associated brain stem centers (Carpenter and Sutin 1983). To the best of our knowledge, the O_2_-sensitivity of CN neurons, however, have not been investigated. One CN nucleus that has been studied in detail relative to its O_2_-sensitivity is the DMNV that forms the ventral half of the cSC and gives rise to the vagus nerve, CN X. As will be discussed in the next section, neurons in the cSC (and thus DMNV) are indeed O_2_-sensitive exhibiting depolarization and the capacity to produce a host of RONS during exposure to hyperoxia (*The O_2_-CO_2_ “gasophilic” cSC*).

Taken together, the above evidence supports the working hypothesis that Sz genesis originates in subcortical areas and involves, at least in part, brain stem CN nuclei and cardio-respiratory centers in the hind brain based on, respectively, the various nonconvulsive S/Sx and complex temporal pattern of cardio-respiration observed during exposure to HBO_2_ (*Integrated cardio-respiratory responses to hyperoxia*). The exact origins of hyperoxic hyperventilation and the concomitant surge in autonomic outflow remains unknown (Demchenko et al., 2007; Demchenko et al., 2011). One likely candidate, as will be discussed next, is the cSC in the caudodorsal medulla (CDM) (Ciarlone et al., 2019).

### The O_2_-CO_2_ “gasophilic” cSC

The NTS and DMNV (cSC) are important brain stem regions for control of normal cardio-respiratory and autonomic functions (Dean and Putnam 2010; Zoccal et al., 2014). Importantly, in the context of cardio-respiratory control, the cSC has been identified as one of several sites of central CO_2_ chemoreception in the mCNS (Nattie and Li 2002; Nattie and Li 2009; Dean 2010; Guyenet et al., 2010; Nattie and Li 2010; Nattie and Li 2012a). It is also one of the subcortical regions (NTS) that had the highest rCBF prior to Sz during exposure to HBO_2_ (Gasier et al., 2015).

We’ve labeled the cSC as “gasophilic” or the “gas loving” region of the CDM because many of the neurons are stimulated during exposure to increased levels of CO_2_ (hypercapnia) and/or O_2_ (hyperoxia) over a physiologically relevant range of P_B_ = 1-3 ATA (Dean et al., 2003). Central CO_2_-chemosensitivity was confirmed by using focal acidosis of cSC neurons to stimulate respiration (Coates et al., 1993; Nattie and Li 2002). Additionally, exposure to systemic hypercapnic acidosis and hyperoxia induces *c-fos* expression in cSC neurons (Teppema et al., 1997). Importantly, intracellular recordings in rat brain slices indicate that exposure to either hypercapnia, hyperoxia, or hypercapnic hyperoxia depolarizes membrane potential (↓V_m_), increases input resistance (↑R_in;_ where membrane conductance ∝ 1/R_in_), and increases the frequency of action potential generation (integrated firing rate, ↑∫FR) of certain neurons (Dean et al., 1989; Huang et al., 1997; Mulkey et al., 2003a; Ciarlone 2016a; Ciarlone and Dean 2016d). Depending on the study and control level of O_2_ used, the proportion of CO_2_-excited neurons encountered in the cSC ranged from 18% to 57% (Huang et al., 1997; Mulkey et al., 2003a; Matott et al., 2014), whereas the proportion of O_2_-excited neurons recorded ranged from 69 to 90% (Mulkey et al., 2003a; Dean and Putnam 2010; Matott et al., 2014).

Not all neurons tested were stimulated by hypercapnia and hyperoxia, however, indicating that O_2_- and CO_2_-sensitivity are intrinsic properties expressed by some but not all cSC neurons. Remarkably, 89–90% of the O_2_-excited neurons in the cSC were also stimulated during exposure to hypercapnic acidosis (Mulkey et al., 2003a; Ciarlone 2016a). When challenged with the combination of hyperoxic hypercapnia the stimulation of ∫FR was greater than either stimulus alone (Mulkey et al., 2003a; Dean et al., 2004; Ciarlone 2016a; Ciarlone and Dean 2016d). These findings indicate that putative central CO_2_ chemoreceptors will be amongst the first neurons in the cSC depolarized by breathing hyperoxia due to their O_2_ (and RONS, *Seizure genesis and propagation*) sensitivity. This may explain why, in humans, the magnitude of the HCVR and the magnitude of the HO_2_VR (0.75 ATA isocapnic hyperoxia) are significantly correlated (Becker et al., 1996).

### Seizure genesis and propagation

But what about CO_2_-excited neurons (i.e., presumptive central CO_2_ chemoreceptors) located in other chemosensitive areas of the brain stem, cerebellum, and hypothalamus? Are they also depolarized early on during exposure to hyperoxia? Currently, we do not know the answer to that question because the O_2_-sensitivity of CO_2_-excited neurons in chemosensitive areas outside the cSC has not been investigated. As discussed in *Conclusions and recommendations for future research on CNS-OT*, the lack of information on how hyperoxia affects central neurons is due to the nearly universal use of “control” hyperoxia when making electrophysiological recordings from neurons in reduced brain tissue preparations and the dearth of neurophysiologists using lower levels of control O_2_ and/or hyperbaric electrophysiology with reduced brain tissue preparations (Dean et al., 2003; Ciarlone et al., 2019).

If CO_2_-excited neurons in other regions of the mCNS, as illustrated in Figure 2 (golden yellow nuclei), are as O_2_-sensitive as those in the cSC (Mulkey et al., 2003a; Dean et al., 2004; Matott et al., 2014; Ciarlone and Dean 2016d), then the CO_2_-chemoreceptor network (Nattie 2000; Nattie and Li 2009; Nattie and Li 2012a) that resides in the brain stem (Li et al., 1999; Nattie and Li 2001; Nattie and Li 2002; Li et al., 2006; Nattie and Li 2010), hypothalamus (Nattie and Li 2012b), and cerebellum (Martino et al., 2007) could conceivably function as one of the O_2_-/RONS-sensitive neural networks involved in Sz genesis; i.e., so called “oxtox trigger nuclei” (Ciarlone et al., 2019).

Appropriating concepts from models of Sz genesis in epilepsy, we have proposed the following theory to explain Sz genesis in CNS-OT (Ciarlone et al., 2019). When breathing HBO_2_, seizures are evoked from trigger zones, specific sites in the mCNS with low thresholds for O_2_-/RONS-induced depolarization in response to increased rCBF and tissue PO_2_ (Demchenko et al., 2001; Demchenko et al., 2003; Demchenko et al., 2007). The increased electrical signaling by O_2_-sensitive neurons residing in presumptive “oxtox trigger nuclei” or zones is propagated along neuronal axons and across electrical and chemical synapses to adjoining neurons arranged in oscillatory, looping-amplifying circuits. Postulated “oxtox trigger nuclei” include the CN nuclei (blue) and CO_2_-chemosensitive nuclei (golden yellow) illustrated in Figure 2. The ventral half of the cSC (green) includes the dorsal motor nucleus of vagus (CN X) and is both CO_2_- and O_2_-sensitive (Dean and Putnam 2010). We postulate that activation of “oxtox neurons” in CO_2_-chemosensitive and CN nuclei initiates the 1) spark or trigger for an eventual Sz plus 2) activation of the concurrent early, abnormal cardio-respiratory responses and nonconvulsive S/Sx that precede an HBO_2_-induced Sz by 2–15 min depending on the specific early “physio-marker”; for example, hyperoxic hyperventilation and bradycardia (Pilla et al., 2013; Stavitzski et al., 2022), increased electrodermal resistance (Posada-Quintero et al., 2022), and various nonconvulsive/noncardiorespiratory S/Sx (Yarbrough et al., 1947; Vann 1988; Donald 1992).

It has been proposed that there are two mostly independent reverberating, looping-amplifying circuits in the mCNS that produce generalized seizures: 1) a forebrain looping circuit (cerebral cortex, limbic system, and basal ganglia), which is responsible for expression of clonic seizures, facial and forelimb clonus, and rearing and falling over; and 2) a brain stem looping circuit (reticular formation of the medulla oblongata, pons, and midbrain, and specific nuclei of the hypothalamus and thalamus) that is responsible for the expression of tonic convulsive seizures and running and bouncing clonic convulsions (Browning 1985; Browning et al., 1993; Kadiyala and Ferland 2017). Importantly, seizures can be produced solely via the brain stem looping circuit (Browning 1985; Browning et al., 1993) as proposed in Figure 2 (green shaded hind brain region). Points of communication between the two circuits enable communication and recruitment of new brain areas into the ictal network to cause more complex seizures (Browning et al., 1993; Ferland and Applegate 1998). Variations in the observed motor behaviors in terms of Sz duration, intensity, and complexity during exposure to HBO_2_ in rodents (Hinojo et al., 2017) may depend on the volume of “oxtox tissue” depolarized during exposure to HBO_2_ and the degree of coordination between the brain stem and forebrain circuits (Ciarlone et al., 2019).

**FIGURE 3:**
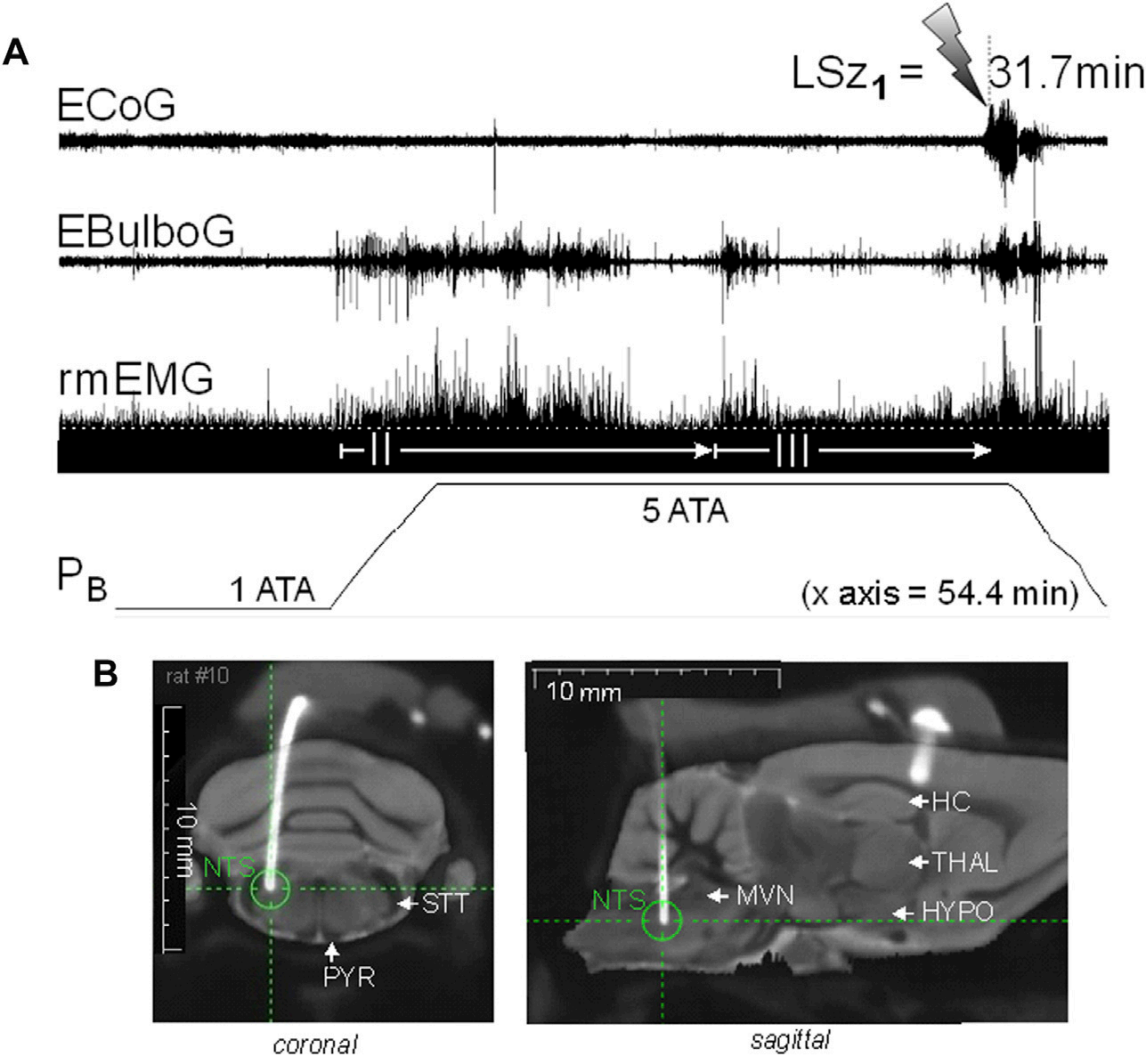
Increasing inspired oxygen from 1 to 5 ATA stimulates neural activity in the DCM and breathing and again prior to corticomotor seizure in a freely behaving male Sprague-Dawley rat. **(A)** Radio telemetry recordings of cortical (ECoG), medullary (EBulboG), and respiratory (rmEMG) activities during exposure to normobaric hyperoxia (1 ATA) and HBO_2_ (>1 to 5 ATA); entire record shown is 54.4min in duration. EBulboG activity increased during compression on 100% O_2_ and remained intermittently active until onset of seizure (lightning bolt). The LSz_1_ measured from 3 ATA to onset of ictal ECoG activity and visible seizure was 31.7 min. EBulboG activity increased 33.6 min before increased ECoG activity. EBulboG activity also correlated with increased ventilation during exposure to HBO_2_. Respiration did not decrease initially when breathing 1 ATA O_2_, thus there was no phase I HO_2_VR as sometimes is the case (see text). Phase II HO_2_VR is evident and quite long while phase III waxes and wanes until surging ∼4–5min prior to Sz. **(B)** Composite CT-MRI image showing location of deep tungsten electrode in the CDM, which was on the border of the NTS and the parvicellular reticular nucleus. HC, hippocampus; HYPO, hypothalamus; MVN, medial vestibular nucleus; NTS, nucleus tractus solitarius; PYR, pyramids; STT, spinotrigeminal tract; THAL, thalamus.

We recently began experiments to test the hypothesis that brain stem nuclei are stimulated electrically early on during exposure to HBO_2_, and furthermore, that brain stem excitation precedes ictal activity in the motor cortex and onset of behavioral Sz (Dean et al., 2022). Figure 3 shows an experiment in an unanesthetized, freely behaving male Sprague-Dawley rat implanted with a radio telemetry module and biopotential leads. In a recent experiment, epidural and deep intracranial telemetric electrodes were used to measure, respectively, cortical motor (electrocorticogram, ECoG) and dorsal medullary neural activities (electrobulbogram, EBulbG) concurrently with respiratory muscle electromyogram activity (rmEMG) before and during Sz genesis at 5 ATA O_2_. The deep intracerebral telemetric method was adapted from that first developed and used in mice (Papazoglou et al., 2016). Notice that EBulboG activity increased during onset of O_2_-compression and in parallel with phase II of the HO_2_VR and remained intermittently active until onset of Sz. During the latter half of phase II, rmEMG activity decreased as expected and in parallel with EBulboG activity. EBulboG and rmEMG activities waxed and waned in parallel during HBO_2_ exposure and finally increased ∼4.5 min prior to Sz. In contrast, ECoG activity did not increase until immediately before Sz. In this example, the Sz occurred 31.7 min after passing through 3 ATA O_2_ on the descent to 5 ATA O_2_. Postmortem analysis using co-registered CT-MRI scans show the deep intrabulbar electrode tip was placed on the border of the NTS and parvicellular reticular nucleus in the CDM. Prior to the HBO_2_ “dive”, this animal was also exposed to 10 min each of normobaric hypercapnia (7% CO_2_ in air) and hypoxia (8% O_2_ in N_2_), which also stimulated EBulboG activity and respiration (not show) (Dean et al., 2022). While these results are preliminary, they suggest that neurons in the CDM and respiration are stimulated concurrently during early exposure to HBO_2_ and prior to onset of cortical ictal activity and observable behavioral Sz.

### HBO_2_-induced stimuli of cSC neurons

At the subcellular level, HBO_2_ could potentially affect neurons in one or more of the following three ways (D'Agostino et al., 2009); 1) directly through its increased partial pressure and narcotic potency, and 2) by the concomitant, separate effect of increased pressure per se; and 3) indirectly through a series of molecular O_2_-initiated L-arginine/catalyzed/reduction reactions that yield the highly reactive byproducts, RONS (Dean 2010).

Molecular O_2_ is thought to have narcotic actions on the mCNS (Hesser et al., 1978), but they are difficult to separate due to the high reactivity of O_2_ to form singlet oxygen, superoxide, and nitric oxide (D'Agostino et al., 2007; Ciarlone and Dean 2016b; Ciarlone and Dean 2016c; Roberts et al., 2017; Hinojo et al., 2021), and its consumption by mitochondrial respiration (Turrens 2003; Larsen et al., 2012). Concerning the effect of pressure per se (baro-sensitivity), certain cSC neurons are depolarized by hyperbaric pressure, but baro-sensitivity does not correlate with either expression of O_2_-sensitivity or CO_2_-chemosensitivity in cSC neurons (Mulkey et al., 2003a; Mulkey et al., 2003b). Moreover, the cellular mechanisms of depolarization and ↑∫FR in cSC neurons for each gas species involve opposing changes in membrane conductance: decreased membrane conductance during HBO_2_, which is presumably a decreased outward potassium conductance that involves free radicals/oxidation, and increased membrane conductance during exposure to hyperbaric helium (baro-sensitivity)^4^ at constant control O_2_, which is likely an increased inward cation conductance that does not involve free radicals (Mulkey et al., 2003a; Mulkey et al., 2003b).

**FIGURE 4:**
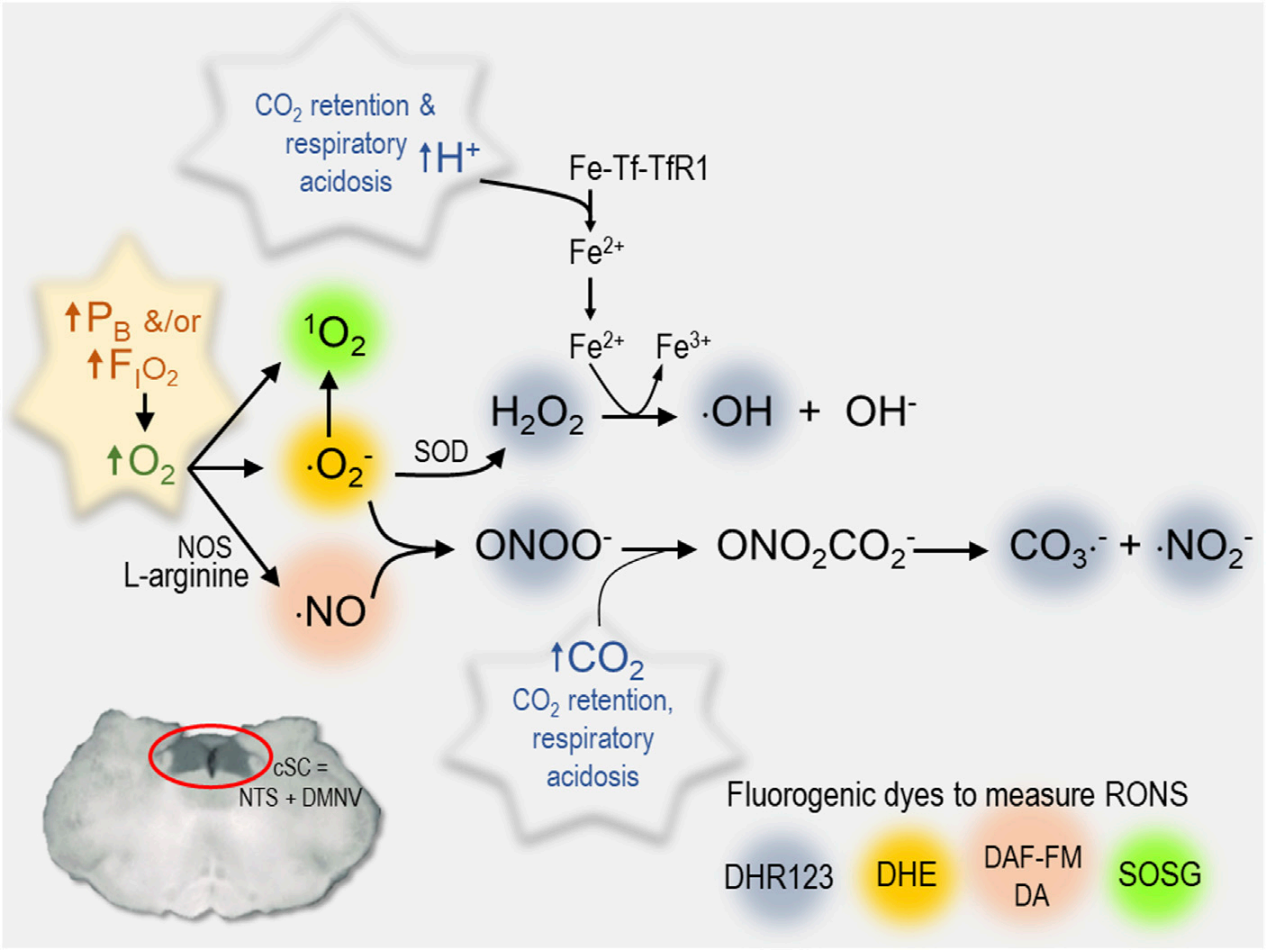
Summary of various RONS measured in cSC cells that increase during exposure to hyperoxia. Each reactive species is color coded to identify the fluorogenic dye used to measure its production in rat brain slices (^1^O_2_, singlet oxygen) SOSG, Singlet Oxygen Sensor Green, 2.5 μM (Roberts et al., 2017) (⋅O_2_^−^, superoxide radical) DHE, dihydroethidium, 2.5 μM (Ciarlone and Dean 2016b; Hinojo et al., 2021) (⋅NO, nitric oxide) DAF-FM DA, 4-amino-5-methylamino-2′,7′-difluorofluorescein diacetate, 5 μM) (Matott et al., 2014; Ciarlone and Dean 2016b); an aggregate of RONS including hydrogen peroxide (H_2_O_2_), hydroxyl radical (⋅OH), peroxynitrite (ONOO^−^), carbonate (⋅CO_3_^−^), and nitrogen dioxide radicals (⋅NO_2_^−^): DHR123, dihydrorhodamine 123, 10 μM) (Ciarlone and Dean 2016c). Other abbreviations used include the following: cSC, caudal solitary complex; DMNV, dorsal motor nucleus of vagus; Fe-Tf-TfR1, transferrin + iron; Fe^2+^, ferrous iron; Fe^3+^, ferric iron; F_I_O_2_, fractional concentration of inspired oxygen; NOS, nitric oxide synthase; NTS, nucleus tractus solitarius; ONO_2_CO_2_^−^, nitrosoperoxocarboxylate; P_B_, barometric pressure; RONS, reactive oxygen and nitrogen species; SOD, superoxide dismutase.

We propose that the third mechanism is the critical stimulus that underlies increased excitability in the cSC during exposure to HBO_2_; that is, reactive species produced by the sequential reduction (electron transfer) of molecular O_2_ (ROS) and production of RNS **(** Figure 4 **)** that, in turn, cause cellular oxidation, lipid peroxidation, etc (Mulkey et al., 2003a; Dean et al., 2004; Thom 2009). For example, neurons in cSC are depolarized by intracellular acidosis during exposure to hypercapnic acidosis (Nichols et al., 2009). Likewise, oxidation of cSC neurons produces intracellular acidification, which stimulates ∫FR of cSC neurons (Mulkey et al., 2004). The acidifying effects of hypercapnic acidosis and oxidation on intracellular pH are additive and mediated in part by inhibition of sodium-hydrogen exchange (Mulkey et al., 2004). The stimulatory effect of oxidation on ∫FR, however, occurs independently of a change in intracellular pH (Mulkey et al., 2004). Further evidence of the importance of oxidation comes from the fact that exposure to the antioxidant, Trolox-C (Vitamin E analog), blocks the excitatory response (i.e., blocks the ↓V_m_, ↑R_in_, ↑∫FR) of cSC neurons during HBO_2_, whereas exposure to either of the chemical oxidants Chloramine-T and N-chlorosuccinimide in control O_2_ mimics the excitatory effects of HBO_2_ on cSC neurons (Mulkey et al., 2003a).

Redox and nitrosative signaling mechanisms are a widely distributed form of intracellular communication in the mCNS in both health and disease (Dröge 2002). Changes in arterial PO_2_ initiate a series of redox and nitrosative signaling reactions that have been identified as fundamental steps in mechanisms of peripheral chemoreception and the HVR (Fernández-Agüera et al., 2015; Gao et al., 2017; Arias-Mayenco et al., 2018), long-term facilitation of respiration (MacFarlane and Mitchell 2008), brain stem control of cardiovascular output (Hirooka 2008; Chan and Chan 2014), and presumably central CO_2_-chemoreception (Mulkey et al., 2003a; Dean 2010; Ciarlone 2016a). Likewise, we predict that O_2_-induced RONS produced during exposure to hyperoxia would activate cSC and other brain stem neurons that possess these redox mechanisms as part of the network’s repertoire of signaling mechanisms that respond to regional changes in tissue PO_2_ caused by variations in rCBF, metabolism and electrical signaling activity. In the presence of HBO_2_, however, these same signaling mechanisms are abnormally stimulated by RONS, which imparts their O_2_-sensitivity and proposed role as “oxtox trigger neurons” (Ciarlone, Hinojo et al., 2019). The specific molecules responsible for cellular oxidation are unknown; however, cSC cells produce a variety of RONS during exposure to NBO_2_ and HBO_2_ (Figure 4). Molecular O_2_ reacts to yield a variety of ROS, including singlet oxygen (^1^O_2_), superoxide (⋅O_2_^−^), and ⋅NO. Superoxide and ⋅NO, in turn, react to produce peroxynitrite (ONOO^−^) that reacts with CO_2_ to produce additional RNS, including nitrosoperoxocarboxylate (ONO_2_CO_2_^−^) that dissociates into carbonate (CO_3_^−^⋅) and nitrogen dioxide (⋅NO_2_^−^) radicals. Respiratory acidosis caused by CO_2_ retention facilitates these redox and nitrosative reactions as summarized in Figure 4. Protons produced by the dissociation of carbonic acid cause transferrin to release iron and catalyze the Fenton/Haber-Weiss Reaction whereby hydrogen peroxide (H_2_O_2_) reacts with ⋅O_2_^−^ to produce hydroxyl radicals (⋅OH) and hydroxide (OH^−^). The cellular consequences of these reactions, include acidosis, nitro-oxidation, and lipid peroxidation (Dean 2010). The synergy between HBO_2_ and CO_2_/H^+^ depicted in Figure 4 is one of the reasons that CO_2_ rebreathing/retention during exposure to HBO_2_ accelerates onset of CNS-OT (Dean 2010). The synergy between O_2_^−^/H_2_O_2_/H^+^ and ONOO^−^/CO_2_ would also explain why the combined effects of HBO_2_ and hypercapnic acidosis are such powerful stimuli of cSC neuronal excitability (*The O2-CO2 “gasophilic” cSC*) (Mulkey et al., 2003a; Dean et al., 2004; Ciarlone 2016a; Ciarlone and Dean 2016d).

To date, fluorescence imaging in real time in rat brain slices shows that exposure to NBO_2_ stimulates production and accumulation of ^1^O_2_ (Roberts et al., 2017), ⋅O_2_^−^ (Ciarlone and Dean 2016b; Hinojo et al., 2021), ⋅NO (Matott et al., 2014; Ciarlone and Dean 2016b), and an aggregate of RONS consisting of H_2_O_2_, ⋅OH, CO_3_^−^⋅ and ⋅NO_2_^−^ radicals (Ciarlone and Dean 2016c); Figure 4. Similarly, exposure to HBO_2_ stimulates ⋅O_2_^−^ (Hinojo et al., 2021). Increasing the PO_2_ of the brain slice superfusate from 0.4 to 0.95, 1.95, and 4.95 ATA O_2_ increases the rate of ⋅O_2_^−^ production that is maintained during the first hour (0.95, 1.95, 4.95 ATA) and second hour of hyperoxia (0.95 and 1.95 ATA). For the highest level of HBO_2_ (4.95 ATA) tested, however, the rate of ⋅O_2_^−^ production decreased during the second hour, which was presumably due to the cumulative effects of oxidative stress and cell death (D'Agostino, Putnam et al., 2007) (*HBO_2_-induced stimuli of cSC neurons*). Interestingly, not all cells tested produced ⋅O_2_^−^ at the same rate during exposure to control O_2_, NBO_2,_ and HBO_2_. Moreover, exposure to ketone salts significantly decreased the production of ⋅O_2_^−^ during exposure to NBO_2_ and HBO_2_ in medullary slices. Moreover, cSC cells that increased ⋅O_2_^−^ production by >25% during exposure to the first hour of hyperoxia (O_2_ sensitive in terms of ROS production) were subsequently inhibited by ketone salts during the second hour of hyperoxia. By contrast, cSC cells that increased ⋅O_2_^−^ production by ≤ 25% during exposure to the first hour of hyperoxia (O_2_ insensitive in terms of ROS production) were not affected by ketone salts during the second hour of hyperoxia. Thus, ⋅O_2_^−^ production in the cSC is stimulated over a physiologically relevant range of hyperoxia, but at varying rates between cells and with varying degrees of sensitivity to ketone therapy. This raises the interesting question of what are the phenotypes of neurons with different capacity of ⋅O_2_^−^ production relative to their sensitivities to CO_2_, O_2_ and other cardio-respiratory stimuli, and neurotransmitters? Taken together, these findings support the hypothesis that the neuroprotective effects of exogenous ketones against CNS-OT in rodents (D'Agostino et al., 2013; Ari et al., 2019; Stavitzski et al., 2021) are due in part to decreased ⋅O_2_^−^ production (and its downstream reaction products) during exposure to NBO_2_ and HBO_2_ (Hinojo et al., 2021). Other possible neuroprotective benefits of ketone therapy during exposure to HBO_2_ include improving mitochondrial function, reducing inflammation, increasing the activity of neurotrophic factors (Maalouf et al., 2009), hyperpolarizing neuronal membrane potential and decreasing synaptic release of excitatory neurotransmitters (Yellen 2008), and increasing the antioxidant capacity in blood serum (Stavitzski et al., 2022). Various countermeasures being studied to determine their effectiveness as mitigations strategies against CNS-OT have been reviewed elsewhere (Ciarlone et al., 2019).

### Interpreting brain slice experiments: PO_2_ (*in vitro*) vs rat brain (*in vivo*)

In the electrophysiology and fluorescence imaging experiments summarized in *Seizure genesis and propagation*, the level of O_2_ used to aerate artificial cerebral spinal fluid (aCSF) media for testing O_2_-sensitivity in brain slice neurons ranged from 0.4 ATA O_2_ (control) to 0.95, 1.95, and a maximum of 4.95 ATA O_2_. To interpret our results in terms of neuronal electrical signaling and RONS production during exposure to hyperoxia, and its relevance to whole animal studies of cardio-respiratory control during HBO_2_, it is important to ask how these levels of O_2_ relate to the physiological range of hyperoxia that humans and animals experience (P_I_O_2_ > 0.2 to 3 ATA O_2_).

Typically, rodent brain slice experiments use 0.95 ATA (95% O_2_ at P_B_ = 1 ATA) as the control O_2_ level (Aitken et al., 1995; Dean et al., 2003). The rationale for using 0.95 ATA O_2_ is that in the absence of hemoglobin, a high FO_2_ is required to ensure adequate O_2_-diffusion from the aCSF into the deeper cell layers of the ≤500 μm-thick brain slice to avoid an anoxic core. No need to worry about anoxia, however, because using 95% O_2_ at room pressure (0.95 ATA O_2_) produces a range of tissue PO_2_ in a 300–400 μm thick brain slice, submerged in aCSF and superfused across both cut surfaces, that is equivalent to that of a rat breathing 2.0–2.5 ATA HBO_2_ (Mulkey et al., 2001; Dean et al., 2003; Garcia III et al., 2010). Therefore, beginning in 2007, we reduced the level of control O_2_ from 0.95 to 0.4 ATA O_2_ and began using 0.95 ATA O_2_ as the lowest test level of hyperoxia. To date, increasing PO_2_ from 0.4 ATA (control) to 0.95 ATA O_2_ (NBO_2_) stimulates RONS production and ∫FR in CO_2_-excited cSC neurons (Matott et al., 2014; Ciarlone 2016a; Ciarlone and Dean 2016b; Ciarlone and Dean 2016c; Ciarlone and Dean 2016d). Baseline ∫FR of cSC neurons is lower in 0.4 ATA O_2_ compared to 0.95 ATA O_2_ but basic electrophysiological mechanisms of excitability are retained for hours (Matott et al., 2014). Thus, exposure to aCSF equilibrated with 0.95 ATA O_2_ is “HBO_2_” in terms of the level of tissue PO_2_ (minus pressure per se) and a stimulant of cSC neurons (Matott et al., 2009; Matott et al., 2014).

Continuing the comparison of tissue PO_2_s in rat brain slices versus the intact rat brain, equilibrating aCSF with 1.95 ATA O_2_ produces a brain slice PO_2_ that is equivalent to that of an animal breathing ∼4.75–5.0 ATA HBO_2_ (Dean et al., 2003). This is also physiologically relevant given that 4 and 5 ATA O_2_ are used to study the pathophysiology of CNS-OT in unanesthetized, freely behaving rodents (D'Agostino et al., 2013; Pilla et al., 2013; Held et al., 2014). By contrast, the highest level of HBO_2_ tested in brain slices, 4.95 ATA O_2_, produces a brain slice PO_2_ equivalent to a rat breathing 6 to ≥7 ATA O_2_ (Dean, Mulkey et al., 2003). Six ATA O_2_ induces seizures very rapidly in unanesthetized, freely behaving rats (Pilla et al., 2013); in fact, Sz occur so rapidly that we no longer use it as a test condition. Six ATA O_2_ is routinely used, however, in anesthetized animals due to the central depressant effects of anesthesia (Demchenko et al., 2012). Following actual bilateral carotid body chemodenervation, exposure to 8 ATA O_2_ blocks the HO_2_VR, which was attributed to oxygen poisoning of brain stem neurons (Cragg et al., 1986). Moreover, as described above, exposure of medullary slices to 4.95 ATA O_2_ after 1 h decreased ⋅O_2_^−^ production in cSC cells, presumably due to the cumulative effects of oxidative stress and cell death (D'Agostino et al., 2007; Hinojo et al., 2021).

We conclude that using 0.4, 0.95 and 1.95 ATA O_2_ in rat brain slices is a useful model for studying mechanisms of cellular O_2_ sensitivity under conditions that relate to those used in intact animal models breathing normocapnic HBO_2_ at 2.0–5.0 ATA.

## Conclusions and recommendations for future research on CNS-OT

CNS-OT is a complex disorder that presents as a variety of nonconvulsive S/Sx of brain stem origin, including a progression of cardio-respiratory abnormalities and “early toxic indications” (VENTID) that culminate in generalized seizures, loss of consciousness, and postictal cardiogenic pulmonary edema. The risk of CNS-OT and its antecedent S/Sx are what limits the use of HBO_2_ in HBOT and undersea medicine. The challenge going forward for the biomedical community is learning how to extend the duration of the safe latent period of HBO_2_ ventilation for safer, longer “dives”, to reap the benefits of HBO_2_, whether on dry land sealed inside a hyperbaric chamber and receiving treatments for wound healing or submerged beneath the surface of ocean for purposes of exploration, national defense, or simply, recreation.

The purpose of this review was to illustrate how the temporal pattern of abnormal brain stem responses that precedes a full blown “oxtox hit” provides researchers a window into the early neurological events underlying Sz genesis. Accordingly, we focused on the early respiratory abnormality, hyperoxic hyperventilation, and the neurons presumed to underlie this response—neurons in the cSC, including putative CO_2_ chemoreceptors in the CDM. The challenge of preparing this article, however, was the relatively few laboratories and studies that have used similar approaches as presented here to study how hyperoxia affects neuronal function in the context of CNS-OT, or in O_2_-sensitivity in general. To address that need, the field would benefit from future research on determining the locations of “oxtox nuclei” to understand the cell phenotypes based on their sensitivity to hyperoxia and RONS, axonal projections, and neurotransmitters. Once this is known, then fundamental questions focusing on the underlying molecular, cellular, and synaptic mechanisms of Sz genesis when breathing HBO_2_ can be investigated. This, in turn, will provide new knowledge into the underlying mechanisms of CNS-OT and which populations of neurons and synapses to target with therapeutic strategies to reduce brain hyperexcitability during exposure to HBO_2_, with the goal of safer, longer “dives” whether made under wet or dry conditions.

While the foregoing approaches have direct relevance for important questions pertaining to hyperbaric and undersea medicine, they also have relevance as a model system for studying all forms of Sz genesis and how the cumulative effects of oxidative stress perturb neurological function in general. In that context, we offer up a recommendation for your consideration; study the effects of lowering the control level of O_2_ used to maintain reduced neural tissue preparations, regardless of the goal of the study. Molecular and cellular studies that employ these important neural tissue preparations, including brain slices, isolated brain stem spinal cord, perfused working heart-brain stem spinal cord, and even cell culture in some cases, are unable to study the effects of hyperoxia because hyperoxia is their control condition. When one of us (JBD) started this line of research in the mid-1990s, he had no choice but to build a hyperbaric chamber to produce hyperoxia in brain slices. Why? Because all brain slice work was done at room pressure using aCSF aerated with 95% O_2_. The only way to produce hyperoxia was to increase P_B_ using a hyperbaric chamber (Dean and Mulkey 2000). Now, we know that control oxygen, at least in medullary and hippocampal tissue slices, can be lowered significantly to 40% and possibly 20% O_2_ and still yield viable electrophysiological recordings to study O_2_- and RONS-dependent mechanisms unencumbered by a background of excessive oxidation and accumulating oxidative stress (D'Agostino et al., 2007; Matott et al., 2009; Matott et al., 2014; Ciarlone 2016a; Ciarlone and Dean 2016b; Ciarlone and Dean 2016c; Ciarlone and Dean 2016d; Hinojo et al., 2021). Assessing the O_2_-sensitivity of popular reduced neural tissue preparations would be a worthwhile investment of time and resources and make an important contribution to the field of O_2_ and redox neurophysiology specifically, and neuroscience more generally.

## Footnotes

1 Other pressure equivalents for 1 atm absolute, and 760 mm Hg or Torr, are as follows: 33.07 fsw; 10.08 msw; 1.013 bar; 101.3 kPa; 0.1013 MPa; 14.7 psi

2
Dripps and Comroe (1947) were previously quoted by Dean et al. (2004); however, it is reiterated here given that their observation is fundamentally important to the theme of this review article.

3 Some of my (JBD) fondest memories working with the undersea medical community have been my interactions with Dr. Chris Lambertsen at the annual ONR-NAVSEA Program Reviews beginning in 2000. Dinners and stories we shared were always entertaining, fun, and thought provoking. During scientific meeting sessions you could always count on C. J. to voice his opinion during the discussion of the investigator’s brief, firmly telling us what we were doing incorrectly, but also always offering words of encouragement. C. J. Lambertsen was a leader in oxygen and decompression physiology and a role model to all who now work in this field.

4 Cellular baro-sensitivity is studied using either hydrostatic compression, which makes studying cell function in real time difficult, or alternatively, using hyperbaric helium (HBHe) as a hydrostatic pressure analog (Dean et al., 2000). Using HBHe is less difficult but technically feasible and has been adapted for real time measurements of neuronal function in rat brain slices during exposure to HBO_2_, including mechanisms of V_m_ excitability and electrical signaling. Helium has an exceptionally low lipid solubility and thus narcotic potency, especially when used at modest levels of hyperbaric pressure, < 5 ATA, as in our studies (Dean et al., 2003).

## Glossary

ATA: atmospheres absolute
CDM: caudodorsomedial medulla oblongata
CN: cranial nerve
CNS-OT: central nervous system oxygen toxicity
CO_3_^−^⋅: carbonate radical
cSC: caudal Solitary complex
CT-MRI: computerized tomography-magnet resonance imaging
DMNV: dorsal motor nucleus of the vagus
EBulboG: electrobulbogram
ECoG: electrocorticogram
FO_2_ and F_I_O_2_: fractional concentration of oxygen and fractional concentration of inspired oxygen
∫FR: integrated firing rate
fsw: feet of sea water
OH: hydroxyl radical
⋅OH^−^: hydroxide
H_2_O_2_: hydrogen peroxide
HBO_2_: hyperbaric oxygen
HBOT: hyperbaric oxygen therapy
HCVR: hypercapnic ventilatory response
HO_2_VR: hyperoxic ventilatory response
HVR: hypoxic ventilatory response
LARU: Lambertsen Amphibious Rebreather Unit
LSz and LSz_1_: latency time to seizure and latency time to first seizure
mCNS: mammalian central nervous system
NBO_2_: normobaric hyperoxia
⋅NO: nitric oxide
⋅NO_2_^−^: nitrogen dioxide radical
NTS: nucleus tractus solitarius
^1^O_2_: singlet oxygen
⋅O_2_^−^: superoxide anion
ONOO^−^: peroxynitrite
ONO_2_CO_2_^−^: nitrosoperoxocarboxylate
P_B_: barometric pressure
PO_2_ and P_I_O_2_: partial pressure of oxygen and partial pressure of inspired oxygen
rCBF: regional cerebral blood flow
rmEMG: respiratory muscle electromyogram
R_in_: input resistance (where 1/R_in_ ∝ membrane conductance)
RONS: reactive oxygen and nitrogen species
ROS: reactive oxygen species
RNS: reactive nitrogen species
S/Sx: signs and symptoms
V_m_: membrane potential
